# Cardiovascular risk and stroke mortality in persons living with HIV: a longitudinal study in a hospital in Yaounde

**DOI:** 10.11604/pamj.2021.40.8.30855

**Published:** 2021-09-02

**Authors:** Liliane Mfeukeu Kuate, Larissa Ange Kwangoua Tchuisseu, Ahmadou Musa Jingi, Charles Kouanfack, Francky Teddy Endomba, Christian Ngongang Ouankou, Leonard Ngarka, Jean Jacques Noubiap, Samuel Kingue, Alain Menanga, Pierre Ongolo Zogo

**Affiliations:** 1 Department of Internal Medicine and Specialties, Faculty of Medicine and Biomedical Sciences, University of Yaounde I, Yaounde, Cameroon,; 2 Cardiology Unit, Yaounde Central Hospital, Yaounde, Cameroon,; 3 Department of Clinical Medicine, Faculty of Health Sciences, The University of Bamenda, Bamenda, Cameroon,; 4 Faculty of Medicine and Pharmaceutical Sciences, University of Douala, Douala, Cameroon,; 5 Department of Psychiatric, Faculty of Medicine of Dijon, University of Burgundy, Dijon, France,; 6 Faculty of Medicine and Pharmaceutical Sciences, University of Douala, Douala, Cameroon,; 7 Centre for Heart Rhythm Disorders, University of Adelaide, Adelaide, Australia,; 8 Department of Biophysics, Medical Imaging and Radiotherapy, Faculty of Medicine and Biomedical Sciences, University of Yaounde I, Yaounde, Cameroon

**Keywords:** Cardiovascular risk, HIV, stroke, death, Cameroon

## Abstract

**Introduction:**

HIV infection is a well-known risk factor for stroke, especially in young adults. In Cameroon, there is a death of data on the outcome of stroke among persons living with HIV (PLWH). This study aimed to assess the cardiovascular risk profile and mortality in PLWH who had a stroke.

**Methods:**

this was a retrospective cohort study of all PLWH aged ≥18 years admitted for stroke between January 2010 and December 2019 to the Cardiology Unit of the Yaoundé Central Hospital, Cameroon. Cardiovascular risk was estimated using the modified Framingham score, with subsequent dichotomization into low and intermediate/high risk. Mortality was assessed on day 7 during hospitalization (medical records), at one month, and one year by telephone call to a relative.

**Results:**

a total of 43 PLWH who had a stroke were enrolled. Their mean age was 52.1 (standard deviation 12.9) years, most of them were female (69.8%, n = 30). There were 25 (58.1%) patients on concomitant antiretroviral therapy. The Framingham cardiovascular risk score at admission was low in 29 patients (67.4%) and intermediate to high in 14 patients (32.6%). Ischemic stroke was the most common type of stroke in 36 persons (83.7%). The length of hospital stay was 11.4 (interquartile range 9.2-13.7) days. Mortality at 1 year was 46.5% (n = 20).

**Conclusion:**

stroke mortality was high in this population of PLWH. Most patients had a low Framingham score, suggesting that this risk estimation tool underestimates cardiovascular risk in PLWH.

## Introduction

The lifespan of persons living with HIV (PLHIV) has improved with the increased access to antiretroviral therapy (ART), thus reducing the occurrence of AIDS-related complications [1, 2]. On the other hand, as HIV becomes a chronic disease, the increased exposure to traditional cardiovascular risk factors raises the risk of atherosclerosis and its complications [3-5], particularly stroke [6]. Moreover, HIV is a risk factor for stroke [7]. Young adults, aged less than 49 years, have the highest risk of HIV infection, and they represent 10-20% of stroke victims [8, 9].

The pathophysiologic mechanisms explaining the occurrence of stroke in PLWH are not fully understood. However, HIV is thought to act directly through vasculitis or indirectly through opportunistic infections, cancers, coagulopathies, or cardioembolic diseases [10, 11]. In addition, HIV infection leads to chronic inflammatory and prothrombotic states that are the basis for cardiovascular diseases [10]. Furthermore, antiretroviral drugs increase the occurrence of dyslipidemia, insulin resistance and lengthen the time of exposure to traditional risk factors [10, 12, 13]. As a result, the incidence of stroke has been rising over the past decades [8].

In Cameroon, with the 90-90-90 targets, the ART coverage rate has increased [14]. In Douala, in 2019, HIV seroprevalence among patients hospitalized for stroke was estimated at 6.6%, representing one in 15 patients [15]. Very few data are available on post-stroke mortality in PLWH. These two conditions together represent a major problem, given the motor disability generated, as well as the heavy additional economic burden. In addition, the clinical picture of stroke in PLWH remains poorly characterized. To improve the prevention and primary management of stroke in PLHIV, we aimed to assess cardiovascular risk before the onset of stroke and to determine post-stroke mortality.

## Methods

**Study design and setting**: this was a retrospective cohort study conducted at the Cardiology Unit of the Yaoundé Central Hospital (YCH), Cameroon. Located in the Centre region, it is one of the largest hospitals in the city of Yaoundé, with a high attendance rate, a varied technical platform, and good geographical accessibility. The YCH, is a referral hospital, who has an emergency, cardiology, a neurology and medical imaging unit where the multidisciplinary follow-up of stroke patients can be done. There is no stroke specialize unit, but patients were consulted and followed by the multidisciplinary team in the cardiology wards. We reviewed the medical records of patients admitted to the Cardiology Unit from 1 January 2010 to 31 December 2019. The one-year survival was assessed using phone calls and medical files.

**Figure 1:**
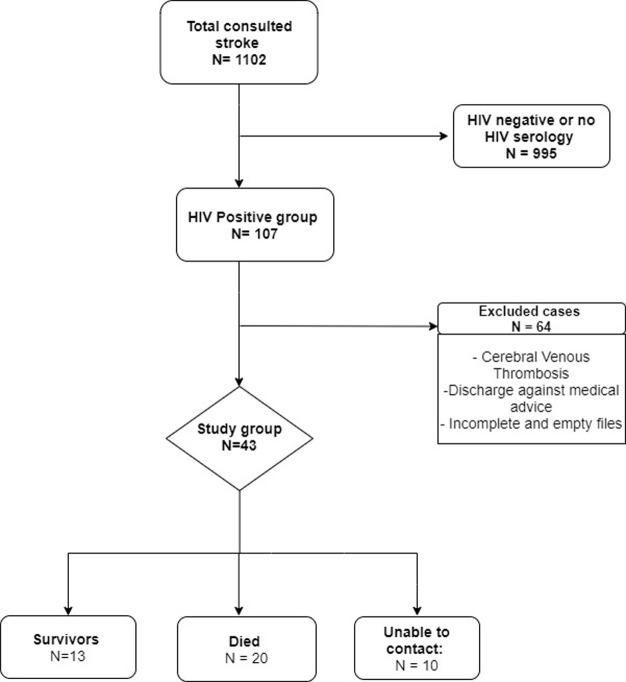
research processflow-chart

**Study population**: we included medical records of HIV-positive patients hospitalized for stroke using the WHO definition, confirmed by brain imaging (Computed Tomography [CT] or Magnetic resonance imaging [MRI]), and aged over 18 years. We excluded records of patients under 18 years of age, those without brain imaging (CT or MRI), those with cerebral venous thrombosis, unreadable or empty patient files, and those discharged against medical advice. Patients with unknown HIV status were also excluded (Figure 1).

**Variables and data collection**: data were collected from the complete records of cardiology admissions in a predefined data collection form

**Exposure**: regarding the exposure, the HIV patients were defined as each stroke patients who has already been diagnosed of HIV or who is under an HAART or who has been diagnosed during the hospitalization. All these elements were taken in the medical records. The characteristics of HIV-positive patients were duration since HIV diagnosis, opportunistic infections, and CD4 counts. Duration since HIV diagnosis was defined as the number of years since the HIV status discovery to stroke occurrence in years. Opportunistic infections were those noted in the file and concomitant with the cerebrovascular event. CD4 counts were those taken during hospitalization or in the 3 months before the stroke and marked in the record.

**Covariates**: socio-demographics, cardiovascular risk factors, laboratories and medical imaging results were abstracted from medical records. The socio-demographic factors included age and gender. The age in years was collected from the patient's file, corresponding to the age recorded at the time of the event. Given the lack of uniformity in the definition of 'young' or 'early stroke onset' and the resulting divergence of definitions, we define 'young' or 'early-onset' stroke as stroke occurring at less than 55 years of age in this study [16]. The cardiovascular risk factors included tobacco smoking, alcohol consumption, diabetes mellitus, and hypertension. Parameters on admission collected for this study included blood pressure, blood glucose, the Glasgow coma scale, and the modified NIH Stroke Score (NIHSS). We considered as PLWH any patient known with HIV, on ART or not; or any patient diagnosed with HIV during hospitalization. The cardiovascular risk score at admission was performed according to Framingham 2008 to assess the overall cardiovascular risk over 10 years. The variables used were age, gender, total cholesterol, HDL-c, treated and untreated systolic blood pressure, smoking, and diabetes. An algorithm based on validated predictors and coefficients (Framingham, 2008) was performed using SPSS software. Cholesterol levels were set to the following values: for total cholesterol<4.1mmol/L and HDLc > 1.6 mmol/L. The variables were recoded and classified into 2 modalities: low and moderate to high. Where low risk was defined as raising FRS < 10%, moderate to high risk was FRS≥20%.The diagnosis of ischemic or hemorrhagic stroke was marked in the record. Ischemic stroke locations were grouped into sylvian (total sylvian, superficial or deep), anterior, and vertebrobasilar (posterior, basilar stem). For hemorrhagic strokes, the locations were classified as deep hemispheric (damage to the grey nuclei: thalamus, caudate nucleus, lenticulate nucleus, internal or external capsule), lobar (frontal, parietal, occipital, temporal) and sub tentorial (protuberance, cerebellum).

**Stroke outcome**: the duration of hospitalization was the number of days between admission and discharge. Mortality was assessed at 7 days, 1 month, and 1 year. Patients medical record were assessed to abstract the vital status at each stage. For 1-year mortality data, patients were contacted by telephone calls. These calls were made and repeated three times within two weeks. Non-reachable persons were considered as lost to follow-up. These were unavailable, unassigned numbers, or cases of people not recognizing the patient.

**Statistical methods**: data were analyzed using IBM SPSS Statistics version 23.0 for Windows (Chicago, Illinois, USA). Continuous variables were described by the mean and standard deviation (SD) or median with interquartile range (IQR), and categorical variables using their frequencies and percentages. or median and interquartile range based on the distribution law. Missing data were replaced by values calculated by imputation to the mean when the percentage of these was less than 5%.

**Ethical consideration**: this study was approved by the Ethics Committee of the Faculty of Medicine and Biomedical Sciences. Data were collected anonymously from medical records. For the patients contacted by telephone, a favorable opinion was obtained after an explanation of the study.

## Results

**General characteristics**: between January 2010 and December 2019, 1102 patients were admitted for stroke, among which 107 were HIV positive. In this group, 43 HIV-positive patients were eligible and included in this study and 64 were excluded (Figure 1). After one year, the vital status was assessed and 20 patients were dead. The mean age at stroke onset was 52.1 (SD 12.9) years. Most patients were female (69.8%, n = 30). The antiretroviral coverage rate was 58.1% (n = 25). Fifteen patients had opportunistic infections diagnosed, among them ten (23.3%) had cerebral cryptococcosis, two (4.7%) had probable bifocal tuberculosis, two (4.7%) cerebral toxoplasmosis, and one pulmonary pneumocystis (2.3%). Tenofovir-Lamivudine-Efavirenz was the most commonly used combination (56.0%, n = 24). The main socio-demographic characteristics are presented in Table 1.

**Table 1:** characteristics of the population (N=43)

Variables	Percentage (%) or Mean (SD)
Female, n (%)	30(69.8%)
Age, mean (SD), years	52.1(12.9)
Age by class, years	
≤ 45	16 (37.2)
45-54	10(23.3)
55-64	9(20.9)
≥65	8(18.6)
CD4, mean (standard deviation), cells/mm^3^	304.1(213.7)
Duration since diagnostic, median(IQR), years	3(0-6)
On ART, n (%)	25 (58.1 %)
Opportunistic infections, n (%)	28(65.1 %)
Hepatitis B, n (%)	2(4.7%)
Hepatitis C, n (%)	1 (2.3%)
Systolic BP ≥140 mm Hg, n (%)	22(51.2)
Diastolic BP≥ 90 mm Hg, n (%)	22(51.2)
Blood glucose ≥ 1.8 **g/dl, n (%)**	1(3.6)
NIHSS≥15, n (%)	7(20)
Glasgow>8, n (%)	40(93)
Comorbidities (known before the stroke)	
Previous stroke, n (%)	9 (21%)
Smoking, n(%)	15 (34.9)
Hypertension, n (%)	31 (72.1)
Diabetes, n (%)	3 (7)
Alcohol, n (%)	21(48.8)

ART: Antiretroviral therapy; BP: Blood pressure; CD4: cluster of differentiation 4; NIHSS: National Institute of Health Stroke Scale

**Cardiovascular risk**: on admission, the time of arrival at the hospital was late with a median of 3 days [1/2-6 days]. Several risk factors were found. Indeed, the majority of PLWH had a low Framingham cardiovascular risk (67.4%, n = 29) before admission. Table 2 summarizes the modified Framingham cardiovascular risk scores at admission and the clinical severity scores.

**Table 2:** distribution of Framingham score

Framingham class	N(%)
Low Risk, n(%)	29(67.4)
Intermediate to higher risk,n (%)	14(32.6)
**Total,n (%)**	43(100)

**Brain imaging**: regarding the type of stroke, 83.7% (n=36) of strokes were ischemic (Table 3). Among the cerebral ischemic strokes, the sylvian territory was the most affected in both groups 88.9% (n = 32). Furthermore, the deep hemispheric location was the most common in hemorrhagic strokes.

**Table 3:** distribution of stroke type and localization according to HIV serology

Localisations	Frequency (N)	Percentage (%)
**Ischemic stroke**	36	83.7
Sylvian	32	88.9
Vertebrobasilar	3	8.3
Anterior	1	2.8
**Hemorrhagic stroke**	7	16.3
Deep hemispheric	5	71.4
Lobar	1	14.3
Sub tentorial	1	14.3
**Associated localization**		
IPH* + VF*	2	4.7
IPH + MH*	0	0

IPH: Intra Parenchymal Hemorrhage; *VF: Ventricular Flooding; *MH: Meningeal Hemorrhage

**Mortality**: despite this, the average length of hospital stay was 11.4 (SD 7.2) days with 27 patients (62.8%) experiencing complications during hospitalization. Infection was the most frequent complication. Infection was the most frequent intra-hospital complication. Mortality at 7 days was 14.0% (n = 6), increasing to 34.9% (n = 15) at 1 month and 46.5% (n = 20) at 1 year (Table 4).

**Table 4:** distribution of in-hospital complications and mortality in the first 7 days, at 1 month, and at 1 year

Complications	Frequency (N)	Percentage (%)
Complications, n (%)	27	62.8
Infections, n (%)	22	81.5
Bed rest complication, n (%)	4	14.8
Convulsion, n (%)	2	7.4
Death, n (%)		
Death < 7days, n (%)	6	14.0
Death < 1 month, n (%)	15	34.9
Death < 1 year, n (%)	20	46.5

## Discussion

This retrospective longitudinal study aimed to assess the cardiovascular risk of HIV-seropositive patients and their mortality after stroke. We obtained the following results. We found that the cardiovascular risk according to modified Framingham was low in the majority of patients (29 patients or 67.4%). Mortality was high and was markedly increasing over time, with about half of the patients who had died at one-year post-stroke. Age is a non-modifiable cardiovascular risk factor. The mean age at stroke onset in this study was 52.1 (SD 12.9) years. This mean is lower than that in the general Cameroonian population (58.7 years) [17]. This may suggest that stroke occurs in a younger population in HIV-positive people than in HIV-negative people. This result is similar to those obtained in Cameroon in 2019 (51.30 SD 10.37 years), and Tanzania 47.2 (SD 14.5) which find stroke in relatively young subjects [15, 18]. HIV-induced chronic inflammation and coagulation disorders increase their risk of stroke [10, 11], and may explain the early age at onset of cerebrovascular events. As a result, stroke in PLWH occurs in a young and active population and poses an economic cost problem.

The gender distribution of the population shows a predominance of women with 30 (69.8%). HIV prevalence among women in Cameroon is higher (3.4%) than among men (1.9%) [14]. In a hospital study in Cameroon, men were more likely to be found among PLWH with stroke [19]. In the literature, there is variability according to sex, with the majority of studies favoring a male preponderance [19-21]. Nevertheless, it is demonstrated that women living with HIV have a higher immune activation in than seronegative women and men [22, 23]. As well, they have an increased likelihood of developing non-calcified coronary plaques, which are biomarkers of atherosclerotic risk. Similarly, women living with HIV have an increased risk of ischemic stroke [24]. Consequently, HIV-positive women, as a group with a high risk of developing atherosclerosis complications, need to be carefully followed up. In addition, improved cardiovascular diseases prevention in HIV-positive women may decrease their risk of stroke.

Cardiovascular risk at admission was low in most patients. The assessment of cardiovascular risk in people living with HIV is ambiguous in the current literature [25-27]. As HIV is considered a vascular risk factor, it would be logical that the cardiovascular risk in people living with HIV would be increased [7]. In fact, on one hand, several studies have shown that conventional cardiovascular risk scores (Framingham, AHA/ACC) underrate cardiovascular risk in HIV/AIDS [25, 28-30]. However, on the other hand, other authors have shown that these standard scores are appropriate to assess cardiovascular risk, or overestimating the risk [26, 27]. For instance, in Cameroon, a study conducted by Noumegni *et al*. showed that the Framingham tool classified more HIV+ patients in the most at-risk group, while our study did not [27]. This discrepancy could be explained by the young age of our study population, with one-third of our sample under 45 years of age. Additionally, the high proportion of women who are known to have a lower score than men could contribute to the global low risk obtained. Moreover, none of our patients were on abacavir, indinavir, or lopinavir/r. The Framingham score does not include non-traditional risks factors in PLWH such as chronic inflammation, immune system activation and its pro-coagulant cascade, and HAART use [6, 31]. According to a Ugandan study, PLWH is twice more at risk of arterial stiffness [32]. Since antiretroviral drugs increase the rate of dyslipidemia and consequently the cardiovascular risk, it would therefore be advisable to construct a more adequate cardiovascular risk assessment score that includes both more sensitive biomarkers of inflammation (hsCRP), immunological markers, and antiretroviral treatment [33]. This relatively low cardiovascular risk in this study may also be attributed to the fact that PLWH is often attending clinics for routine check-ups, which usually include clinical and biological evaluation to detect cardiovascular risk factors at an earlier stage.

The performance of brain imaging is an important part of the management of stroke patients. We excluded cases with unmarked or inconclusive CT scans or MRI results. PLWH had high proportions of ischemic stroke. In the majority of studies performed, PLWH had more ischemic strokes [18, 20, 21, 34]. This result is similar to those found in Cameroon, Tanzania, and Malawi [15, 18, 20, 35]. Several factors would justify the occurrence of ischemic stroke at the expense of hemorrhagic stroke. HIV infection may predispose an individual to arterial and venous thrombosis due to protein C and protein S deficiencies. Certain infections including Mycobacterium tuberculosis (TB), Cryptococcus, Varicella zoster virus, and syphilis may be implicated because of the immunosuppression caused by HIV. Several reviews on stroke and HIV, advise actively searching for tuberculous meningitis and other opportunistic infections in PLWH who have had a stroke [10, 11]. Carotid locations (sylvian and anterior) were the most common in both groups. This result is similar to that found in a prevalence study with 70.8% of carotid locations for ischemic stroke in PLWH [15]. Therefore, in the case of ischemic stroke, infectious causes should be investigated. For hemorrhagic stroke, hypertension was the most frequent cause with 3 patients (42.9%). This is similar to the results found in Ghana, showing that hypertension was the main cause of cerebral hemorrhage in PLWH (85.7%) [36].

The duration of hospitalization was 11.4 (SD 7.2) days. This is longer than that found in the general Cameroonian population after a stroke (8.56 [SD 6.35] days). This result is similar to those found in the literature with a length of hospitalization of 9 (10-6), 10 (SD 8) days, and 10.3 (IQR 8.2,-12.5) days respectively in Cameroon, Thailand, and Tanzania [10, 13, 15]. The mortality rate at the end of the first 7 days was 14.0%, which is similar to the mortality rates in HIV-negative individuals as described by Dewan *et al*., but lower than that of Ekeh *et al*. (26.7%) [37, 38]. This difference can be explained by the improved management of HIV-positive patients and increased surveillance of the occurrence of infections during their post-stroke follow-up. Indeed, the acute phase of stroke is a crucial period for survival and functional recovery. Thus, appropriate management can reduce the mortality rate during this period. Therefore, a good follow-up of PLWH is necessary.

The one-month mortality rates (34.9%) after stroke were similar to those in the general population in Nigeria (33.3%) and HIV-positive patients in Tanzania [18, 38]. Achieving similar mortality rates as the general population may indicate that HIV-positive patients have a unique protective effect. In fact, due to their age, they can recover faster from neurological damage and therefore have similar clinical severity. Moreover, no opportunistic infections were present in 65.1% of the patients and 58.1% were already on ART. These features may suggest that most of the patients were immunocompetent and therefore similar to the general population. Several studies have addressed this issue, but the conclusion is mixed. Indeed, in a study of the Thai national database, which did not include whether HIV-positive patients were on ART, the work described a high in-hospital mortality rate among PLWH, as well as numerous complications [21]. However, this mortality rate is still higher and may be due to inadequate emergency management. Indeed, the use of rt-PA in acute ischemic stroke is not available in our study site, and neurosurgical management for hemorrhagic stroke remains costly for patients in a developing country. Improved access to both financial and material resources for these innovative techniques could significantly reduce mortality, bearing in mind that the most affected population is made up of workers (active people) who are sources of household income.

As follow-up after stroke is an important aspect, several studies have evaluated mortality after one year. Indeed, in this sample, it was 46.5%, supporting those found in Malawi in 2012 with 40.1% mortality in PLWH [20]. This Malawian study described there were no significantly difference between PLWH and HIV negative person one year after stroke occurred. However, these figures are elevated compared to those of the general population in the world [8], in Africa [39], and in Cameroon [40]. The global burden of diseases estimated at 36.2% the stroke mortality rate in the world, while an African systematic review estimated it at 33.2 (95% CI: 23.6-44.5) [8, 39]. Additionally, in Cameroon, in two referral hospitals, a study reported 32.7% of deaths at one year in 2015 [40]. This high mortality rate can be due to our socio-economic context where the health insurance system is poorly implemented and the entire financial burden of stroke relies on victims and their families. Hence, access to rehabilitation, home care and regular medical checkup constitute an additional expense for the affected households. Consequently, there is a serious need of poststroke rehabilitation, since the majority of patients may not have access to acute therapy and may have at the end severe disability.

Moreover, in our study population, which is predominantly under 55 years, ischemic stroke is most common, the overall mortality remains high compared to other studies [41, 42]. In fact, Varona *et al*. in 2011, found about 4-5% of mortality among young people a year after an ischemic stroke, while Loes *et al*. in the Netherlands, noticed 2.4% [41, 42]. This difference may be due to better monitoring, reduction and management of risk factors in high-income countries compared to our study setting. Thus, identifying the risk factors of death in PLWH would enable us to take steps to improve treatment, survival, quality of life, and return to work in our context.

This study has several limitations. First, due to the use of medical records for the study, the inclusion of cases was limited to the files of patients who were already diagnosed or were diagnosed during the hospitalization of HIV infection. Thus, some patients who were not included might have been HIV-positive but not diagnosed. Similarly, given the health care cost associated with stroke, some patients may have been under-diagnosed due to failure to perform or unavailaibility of brain imaging and other tests requested. Moreover, by its retrospective nature, the absence, nonexistence, and poor representativeness of the archives for certain periods have reduced our sample size of the study knowing that the way in which they were written and store varied from one year to another. Indeed, the archives are not computerized in the Cardiology Unit. In addition, recruitment was done only on hospitalized cases; we did not include patients who died before admission and those who remained at home, so we may have missed some cases, it was a hospital based study. Therefore, all these elements could explain our relatively small sample size. However, our study paved the way for future large-scale case studies on stroke in HIV patients.

## Conclusion

Mortality was high in this population of PLWH, with half of them who had died a year after stroke. There is a need to improve stroke prevention and management in these patients. Furthermore, most of these patients with stroke had a low Framingham score, suggesting that this risk estimation tool underestimates cardiovascular risk in PLWH. Cardiovascular risk prediction could be improved by the addition of biomarkers to the current risk stratification schemes.

### What is known about this topic

- HIV is an independent cardiovascular risk factor through chronic inflammation and antiretroviral therapy;
- Stroke in young subjects occurs in a population at risk for HIV.

### What this study adds

- Stroke mortality is high in PLWH in our setting;
- This study provides a global overview and sets the stage for future stroke studies in PLWH, especially women;
- The Framingham score seems inadequate to assess cardiovascular risk in PLWH.
